# 1. Journal of Health. Periodical. Philadelphia, 1829. 2. The Catechism of Health; or Plain and Simple Rules for the Preservation of the Health and Vigor of the Constitution, from Infancy to Old Age

**Published:** 1831

**Authors:** 


Art. VIII. 1. *Journal of Health.* Periodical. Philadelphia, 1829. 2. *The Catechism of Health; or Plain and Simple Rules for the Preservation of the Health and Vigor of the Constitution from Infancy to Old Age.* p. p. 195. Philadelphia, 1831.

'Health! Health! Health!' was the exclamation of a venerable female friend, who entered our parlor, (the appropriate place for reviewing pretty books,) at the moment when a beautiful copy of the 'Catechism of Health' was laid open for the purpose which now occupies us.

According to our fair *quintagenarian*, the last century was distinguished for producing *good health*: the present, for producing

*good books on the art of health;* and it was quite apparent from her manner that she preferred the former. She seemed even to doubt, whether the numerous works on Hygiene, are productive of any solid advantage to society; and more than once broadly intimated, that the tendency of the whole, is to subject to artificial rules, that which can be safely confided only to the instincts, appetites, and experience, of each individual. She insisted, that those who gave least attention to the preservation of their health, generally enjoyed it most; that man was created for objects, the industrious pursuit of which, will generally give him good health; that it is a natural, not an acquired condition; that an individual may, to be sure, have excellent health, while observing the minute and multiplied precepts of the Journal and Catechism, but it by no means follows, that he would not have been equally well, if these entertaining books had never been written; that such works, like treatises on domestic economy, can but embody the experience of the world, in reference to which each individual might safely be left to appropriate to himself, such a portion as his peculiar situation might require; finally, that to 'live, move, and have our being, beneath a written codeof hygienic laws, is to be subjected to a new dominion, in addition to those which society had already imposed.' We secretly wished for the aid of our learned friends, the Messrs. Editors and Authors, whose principles were thus assaulted, but determined to make as grave a defence as possible, when the animated assailant suddenly took her leave, and left us to reply with the pen instead of the tongue.

We admit, then, that much depends on a good natural constitution, and still more on exposure and hard labor in the open air; but all have not good constitutions, and, in civic life, the majority are placed under circumstances in no degree favorable to great bodily vigor.

The restraints imposed upon them are numberless; their whole existence is, so to speak, artificial; most of the causes, both moral and physical, which act upon them, have an unfavorable bearing; some organs are overworked, others languish in sloth; this is kept in perpetual irritation, that in stagnation and torpor; the temptations to improper indulgences of every kind, are greatly multiplied; the appetites are bribed and pampered; the dominion of fashion is mad and tyrannical.— Hence new infirmities and diseases unknown in the country, are generated; children are born with frail constitutions; and their native tendencies to disease, await but the action of slight exciting causes, to rouse them into fatal energy.

Thus surrounded and put upon, by the enemies of health and long life, the inhabitants of populous cities, must either yield themselves up as willing victims, or study and practise the arts of defence and preservation. To aid them in these respects, is the design of the works now before us. They are far, however, from being limited, in their objects and value, to the inhabitants of cities, though most adapted to those, as by them such works are most needed.

The *Journal of Health*, published twice a month, was commenced in Philadelphia, nearly two years ago, and is ably edited, by two of the most learned and estimable physicians of that metropolis. We have too long neglected to give it a passing notice, but are happy to know, that *even* without our backwood's testimony to its merits, they have become known and acknowledged over the whole United States. Its scope is comprehensive; and, in general, the execution is such as to render it at once entertaining and useful. It abounds in pertinent extracts from writers of celebrity, both ancient and modern; and its notices of current works are given with sound judgment; while the original articles, not a few in number, are written with a degree of tact and spirit, that must ensure them, what but few of the lucubrations of the day, perhaps, receive—a perusal.

We shall extract two or three specimens at random.

### BEDS.

"Many, perhaps, who rank 'the downy couch' high upon their list of indispensable comforts, will be surprised to learn that writers on the means of preserving health have, almost unanimously, included it among the means calculated to rob the system of its due degree of vigor.

In youth especially, feather beds, like every other species of luxury, by causing a premature development of the system, without strength proportionate to the rapidity of its growth, often lay the foundation for many of those diseases by which multitudes are consigned to an early grave.

Even in persons of maturer years, by the undue degree of heat they accumulate around the body during sleep, and the profuse perspiration thence induced, in the milder seasons of the year, their tendency is to enervate the constitution, and lay it open to serious impressions from trifling degrees of cold.

A mattress, composed of some soft and elastic material, as hair or moss, ought, therefore, to be invariably preferred. The latter, with a proper amount of covering, will be found sufficiently warm for health and comfort, during even the severest nights of winter.

On a former occasion, we attempted to show the susceptibility of infants to the influence of cold; hence, it might be supposed that for them, a feather bed would be the most appropriate; and this opinion has been attempted to be supported by a reference to the instinct of animals, among which 'all that bring forth a feeble offspring, prepare a soft bed for their reception—brood them with their wings, or clasp them to their bosom for the sake of warmth.' It is certainly true, that during infancy a greater degree of warmth is at all times demanded than is necessary, or would be proper, in after life; but, as an infant should never be allowed to sleep alone, it can always be preserved of a sufficient temperature, without having recourse to the doubtful expedient of subjecting it to immersion in a bed of feathers.

Dr. Darwin has advised that young children 'should not lie on *very hard* beds, as it may occasion them to rest on too few parts at a time, which hardens these parts by pressure, and prevents their proportionate growth.' A bed, such as is here described, would most undoubtedly be improper at any period of life. There is a very material difference, however, between a soft and elastic mattress, and a bed so hard as to occasion uneasiness to the parts with which it is in contact. From sleeping on the former, even the most delicate need not be deterred, by any apprehensions of the injurious consequences to which the doctor alludes.

If ever feather beds be admissible, it is in the case of the aged, who are nearly as susceptible to the influence of cold as infants; to such, therefore, a warm bed is often a matter of indispensable comfort.

Feather pillows are not less injurious than feather beds. By preserving the head of an immoderate warmth, they are apt to induce catarrhs, and, in the young, may become the remote or exciting cause of inflammation in the ear—eruptions—pain of the head, or even more serious diseases. For the same reason, all coverings for the head at night, excepting in the instance of females who are accustomed to wear a cap during the day, are productive of bad effects. Children, therefore, of both sexes, should be accustomed from an early age, to sleep with the head bare—the covering with which nature has, in general, so plentifully supplied this portion of the body, being amply sufficient to protect it from cold.

After what has been said above, upon the injurious tendency of subjecting the body to an undue degree of heat, during the period of repose, cautions against an excess of bed clothes would appear unnecessary. It is all important that the body be covered with a sufficiency of clothing to preserve it comfortably warm; and this may be effected during health, and in individuals accustomed to exercise, by fewer blankets, coverlets, and *comfortables*, than many are accustomed to pile upon the bed.

So injurious is an excess of heat, during repose, esteemed by Dr. Beddoes, that he has advised, and with great propriety, that young persons, especially when they present symptoms of languor and debility, or complain of unrefreshing sleep, should be examined when in bed, 'and if found too warm, awakened without compunction.'—The bed clothes should then be thrown off, 'or if the dry heat of the surface be considerable,' he adds, 'it will be best to walk up and down the room, in a dress so contrived as to guard the extremities from chill, while it permits the residue of the body to be freely ventilated.' Cool rooms, mattresses, and light bed clothes, will, in all cases, prevent the necessity of having recourse to the expedient here directed.

A proper night-dress is an object of no little importance.—A loose flannel gown for winter, and one of muslin for summer, will be found the most proper, more especially for children. No part of the clothing worn during the day, ought, in fact, to be retained at night.* Whatever dress is adopted, it should be free from every species of ligature, particularly at those parts which encompass the neck or the extremities. This is an all-important caution, from a neglect of which serious injury has often resulted.

Closely shrouding a bed with curtains, is one of those numerous instances in which the requisitions of fashion are found to be opposed to health. By preventing a free circulation of the air, they oblige the individual who reposes within them, to breathe an atmosphere vitiated by repeated respiration. They become receptacles for fine particles of dust, which are liable to be inhaled during sleep, whenever disturbed by the motion of the curtains or of the bedstead: this alone, according to Willich, is a cause to which many young persons may refer the first development of a consumptive attack.

Equally pernicious is the practice of sleeping with the face enveloped in the bed clothes, as well as that most ridiculous custom, so prevalent in this country, of suspending a curtain over the front of an infant's cradle.

Their own feelings might be supposed sufficient to induce all to assume in bed, that position, in which every portion of the body will be left the freest from constraint: yet in the case of children, some cautions may be necessary, in order to prevent an awkward position from being indulged in, calculated to produce a prejudicial effect upon the symmetrical growth and perfect development of the system. Hence

* Those in particular who are accustomed to wear flannel, will find it advantageous to dispense with it whilst in bed—or to exchange it for an under-dress of cotton.

it is prudent, when young persons lie upon their backs, to reduce the size of the pillows, in order to guard against a contortion of the spine; while lying on the side requires pillows sufficiently large to fill up the spaee between the head and point of the shoulder. A constrained position, if it have no other bad efiect, is a certain preventative to sound and refreshing sleep.

Beds should never be placed upon the floor: it is well known, that in all apartments occupied by living beings, the inferior portions of the atmosphere are always the most impure. The most wholesome situation for the bed, is in the middle of the room, and raised some feet from the floor. From the vitiated state of the atmosphere immediately above the latter, and the great importance of a free ventilation, the practice of placing the children's bed beneath another bedstead during the day, cannot be too severely reprobated."

---

"It is a difficult task to so lay down any general principle, and enforce it by examples in detail, that the perverse ingenuity of some cannot find omissions, which they are fain to regard as exceptions in their favor. We have repeatedly, in this Journal, entered our solemn protest against the sin of drunkenness, and pointed out with some care, the masks its votaries put on, to evade the reprobation with which the vice, in its grosser forms, is universally stamped. We have indicated the various means by which at first the health, and afterwards the disposition of children are ruined, by an early indulgence of their appetites; and reprobated the false method, devised in well-intentioned, but mischievous ignorance, of attempting to restore lost strength by domestic prescriptions, recourse to wine bitters or home-brewed liquors. Our efforts have, we are well assured, been attended with some success; and we are encouraged thereby to persevere and continue to point out the various malpractices, by which the body's health and mind's peace are slowly, but surely destroyed.

The evil to which we would now direct the attention of our readers, and entreat them to abandon or shun, as the case may be, is not confined to either sex exclusively; nor is it one for which the inconsiderateness of youth, or the infirmities of age, can be pleaded as palliation. The enjoyment which it brings is solitary, as that from dram-drinking itself, and in its consequences, if possible, still more pernicious. It is, in fact, dram-drinking on a small scale, and in a more fashionable, and, as it is thought, scientific manner. It is a concentrated poison, not jovially quaffed from the glass and the bowl amid songs, and joyous shouts; but carefully meeted out in drops by the idle and luxurious man, who has lounged away his day in listlessness at home, in place of courting occupation and enlivenment by active exercise in the open air; or by the belle, whose pallid face and sunken eye, show the exhaustion of the midnight assembly and dance. They dare not hope for, they are sure they cannot obtain, the sweet sleep which follows industrious labor and useful exertion; but they must forget themselves; the day had for them sufficient horrors, without a wakeful night redoubling the store. What then, say they, remains for them to do, if not to take their accustomed number of drops of laudanum, or some equivalent stupifying solution. They who are afraid to meet the summer's heat or winter's ice—whose nerves are too feeble to bear the slight motion of a carriage, or the shortest ride on horseback—and to whom pain is dreadful even in idea, have no hesitation in thus nightly swallowing a poison, each drop of which, causelessly taken, brings with it more bodily uneasiness and mental torment, than the longest day to the lashed galley slave. They may sleep the sleep of stupefaction, or dream themselves in paradise; but when they awake, 'fear, sorrow, suspicion, discontent, cares, and weariness of life, surprise them in a moment, and they can think of nothing else: continually suspecting, no sooner are their eyes open, but this infernal plague of melancholy seizeth on them and terrifies their souls, representing some dismal object to their minds, which now, by no means or persuasions they can avoid."

However repugnant to our feelings as rational beings may be the vice of drunkenness, it is not more hurtful in its effects than the practice of taking laudanum. Disgusting and repulsive to the eyes of others, and injurious to the indulgers in it, as is the chewing of tobacco, it is not more censurable, nor so much to be dreaded in its consequences, as the habit of chewing and swallowing opium, to mitigate unpleasant feelings, or remove melancholy. Some have habitual recourse to laudanum or opium, to relieve pain and uneasiness, caused usually by the undue gratification of their appetite. Let them learn, that there is no example on record of any agent used for medicinal purposes, in particular diseases, or alarming emergencies, which has on these occasions a direct, controlling, and sanitary power, that will not, when persistently used, become noxious to the animal economy, and poison all the springs of life. It is thus with wine, alcoholic liquors, opium and laudanum, and the various tinctures and cordials of which opium is the basis: it is thus with all the vegetable bitters and mineral tonics without exception. All the powders and cordials which have been recommended for the cure of gout, have invariably, when taken for any length of time, destroyed the digestive powers, enfeebled the brain and nervous system, and often brought on dropsy, palsy, and apoplexy. A physician, after due deliberation and much counsel with himself or medical friends, will prescribe mercury, bark, opium, or perchance arsenic, for the cure of the violent and dangerous malady under which his patient is laboring at the time; and his efforts will often be crowned with success. But let this patient, of his own accord, or under the pestilential influence of domestic or empirical advice, use any of these articles for a length of time, and for one uneasy symptom, which he wished removed, ten will take its place; and his constitution will be so broken down, that even his first successful adviser and medical friend, can now be little more than a melancholy spectator of remediless decay. This is not the language of exaggeration or speculative fear. We speak from a full knowledge of the facts. We repeat it—the person who gives into the habit for weeks, (he may not reach to months, or if he pass these, his years will be but few and miserable,) of daily measuring out to himself his drops of laudanum, or pills of opium, or the like deleterious substance, call it tincture, solution, mixture, potion, what you will, is destroying himself as surely as if he were swallowing arsenic, or had the pistol applied to his head. The fire of disease may for a while be concealed—he may smile incredulous at our prediction; but the hour of retribution will come, and the consequences will be terrible.

Besides, who are the unfortunate creatures, who, in impious despair, destroy themselves by poisoning with opium or laudanum? The very same who had long been in the practice of using it as a soother and a balm: as a means of procuring repose after the languor of idleness, or the perturbations of vice. Miserable resource from care or grief! to stupify one's self with such a drug for a few short hours, only to awake in renewed despondency, with a mind paralyzed and unfitted for the commonest duties of life. The countenance of the unhappy victims of the practice, reveals too painfully to an observant eye their condition. The expression is more haggard, and the features more distorted, than even from common drunkenness, and produce on others a mingled feeling of pity and fear. The humid lustre of the eyes is exchanged for a dull, turbid, and dejected appearance of this organ, which is sunk in its orbit, the rounded cheek, once flushed with the glow of health, is now pale or leaden, and the corners of the mouth no longer raised into ready smiles, have a downward direction, indicative of suffering, alternated with listlessness and apathy. The moral nature is not less fearfully changed than the physical All manly resolution is fled: to think is too great an effort: the sight of distress elicits childish grief, without furnishing sufficient incentive to its relief or mitigation. Not very different, in fine, is the confirmed opium-taker from the torpid animal warmed into motion by artificial heat: it twists itself about, attempts some gambols, or with impotent malice, tries to bite and annoy those near it. But in a few minutes, the stimulus of heat is gone, and it sinks once more into torpidity."

We are tempted to make another extract, from the hope of recalling the attention of the profession to the great value to invalids of horseback exercise, so rapidly falling into disuse in these days of machinery.

### RIDING ON HORSEBACK.

"In some of the former numbers of this journal, we have taken notice of those species of exercise which are within the reach of almost every class of society—of the poor as well as the rich; we proceed now to the consideration of others, which, as they involve considerable expense, must necessarily be confined in our cities at least, to individuals in affluent circumstances.

First upon the list, is riding on horseback: one of the most manly, innocent, and useful kinds of exercise of which any one can partake, and by the use of which, the invalid has not unfrequently been surprised into health.

Bishop Burnet, in one of his works, expresses his surprise that the lawyers of his time, enjoyed, in general, better health, and were longer lived than individuals of other professions. Upon consideration, he was led to attribute this entirely to their being obliged to "Ride the Circuit" almost constantly, in order to attend the various courts held in the different parts of England; and which they were accustomed to do chiefly, if not entirely, upon horseback. It is certainly very reasonable to suppose that this circumstance may have had a very beneficial influence upon their health, and have aided not a little in prolonging their lives.

It has been supposed by some, that riding is a more salutary exercise, and ought to be preferred to walking. This, however, is by no means the case, under ordinary circumstances. Riding, occasionally is confessedly a very powerful aid to health; as an ordinary means of exercise, it is, however, inferior to walking—the latter being in general much better adapted to promote an equal distribution of the fluids to the different parts of the body—to impart to the fibres their due degree of elasticity, and in this manner to augment the health and strength of the whole system. In those cases, however, in which a debilitated constitution or the presence of disease prevents a sufficient amount of exercise from being enjoyed on foot, riding on horseback is to be preferred. As a general rule, it may be said, that walking is best adapted to the preservation of health—riding to the relief of chronic disease—In active diseases neither of them are advisable.

By the dyspeptic, and those predisposed to pulmonary consumption, in particular, riding on horseback is an exercise which should never, if possible, be neglected.

Though we are not prepared to assert with Sydenham, Cullen, and some other physicians, that "horse exercise is an effectual antidote to the consumption" after it has once become seated in the lungs; yet we have seen sufficient to convince us, that when, from predisposition, the disease is to be feared, or the individual already experiences its rapid approach, riding on horseback, persevered in daily for a length of time, in connection with a well regulated diet and proper clothing, is the best, perhaps the only means by which its attack can be avoided, or its further progress completely arrested, and a comfortable existence enjoyed for a series of years.

In riding for exercise, or to preserve health, eight or ten miles a day are sufficient; but for the purpose of restoring health, these little excursions will avail but little. It is not from the fashionable half hour's ride, morning and evening, in which the same ground is travelled over, for the most part, every day, and the surrounding objects cease to interest, from being too frequently presented to the view, that the invalid is to anticipate any decidedly beneficial effects. To produce these, hours must be daily spent on horseback—the mind must be free from depressing or intense reflections; and in the company of a judicious and agreeable companion, such portions of the country should be visited, in which the novelty or beauty of the scenery is calculated to interest the mind and elevate the spirits. Long journeys have hence, with great propriety, been recommended to invalids. To such as can afford it, a ride at a proper season of the year, to some one of our remote watering places, or springs, presents a very excellent means for recruiting health. Let not the indolent and irresolute object to this latter jaunt in consequence of the distance, or the roughness of the road, over which, in many instances, they would be obliged to travel. These circumstances are to be viewed rather in a favorable than an unfavorable light. We can conceive of but little benefit that would be derived, in the way of exercise from a journey of any distance, upon a rail road, and in one of the newly invented self-propelling cars.

Against a species of passive exercise, in which many are fond of indulging, we beg leave here pointedly to protest—we allude to the practice of lounging on horseback—in other words, moving at a snail's pace, over a smooth road, with the external senses but half awake, and the mind in a state approaching to complete apathy. It is true, that the individual who practises this *gentle* kind of riding, may enjoy the benefit of the fresh air; but as to bodily exercise, he experiences even less than the child does upon his rocking horse, or the rustic in his favorite swing upon the barn-yard gate.

Exercise upon horseback should be taken, during summer, in the cooler portions of the day—in general, it is better adapted to clear weather in the more temperate seasons of the year, than to those seasons accompanied by extreme heat or intense cold."

On the whole, the Journal of Health is a work that merits the approbation of the profession, and deserves general patronage. Its learned editors have shown, that a great variety of amusing and instructive topics may be conveniently brought within its limits; and it is by this extensive association, that much of the interest which it has inspired, has been produced and sustained. It is the friend of knowledge and virtue, as well as of health; it presents many specimens, both extracted and original, of fine writing, and as a family periodical, may be ranked with the most useful of the day.

*The Catechism of Health*, like the Journal, is anonymous, but no one, familiar with both, can fail to perceive, that they have a common origin. The Catechism is strictly didactic, and totally divested of all references to authorities, and of every extraneous embellishment. It is, in fact, a rigid elementary and practical treatise on hygeine, in the form, as its title imports, of questions, which, in general, are asked and answered in such manner, as to sustain the attention of the reader, and render the various subjects intelligible to the humblest capacity; while the information communicated, is of so much importance to all classes of society, not even excepting the Faculty of Physic, that we trust it will be speedily and widely disseminated. The Catechism is, indeed, a valuable family book; and should be classed with those, which children and young persons, at home or in school, should be required to study, as they study other elementary works in the various departments of human knowledge. Many have been the treatises of health with which the world has been favored or oppressed; but we know of none, in which so great a number of useful maxims are condensed into so small a space, or arranged in an order so convenient and natural. But we shall let the little book speak for itself.

FOOD.

Q. 243. When ought man to partake of food?

A. Whenever he feels a natural appetite for it.

Q. 244. What is meant by a natural appetite?

A. That inclination for food which occurs in a healthy individual accustomed to daily exercise in the open air.

Q. 245. Of what kind of food ought man to partake?

A. Of plain and wholsome food, simply cooked.

Q. 246. Ought it to consist of vegetables or the flesh of animals?

A. Of a proper mixture of both.

Q. 247. Of which should it consist principally?

A. Of Vegetables, particularly in summer, and in warm climates.

Q. 248. How much ought an individual to eat?

A. Only so much as to satisfy his natural appetite.

Q. 249. Can an individual enjoy perfect health on animal food alone?

A. He will at least enjoy less perfect health than on a proper intermixture of animal and vegetable food; or by livlng entirely upon the latter.

Q. 250. Is it proper to eat with haste?

A. No; every portion of the food should be fully chewed before it is swallowed.

Q. 251. If our food be not properly chewed, what is the consequence?

A. It cannot be fully digested by the stomach; hence it overloads the latter, does not yield sufficient or proper nourishment to the body, destroys the natural appetite, produces uncomfortable feelings and disease.

Q. 252. In what manner is an unnatural and inordinate appetite for food produced?

A. By partaking of a great variety of food, or, of that which is richly cooked; by rich sauces, high seasoning, and by the use of wine and stimulating drinks at meals.

Q. 253. Is not a man's health and strength always in proportion to the amount he eats?

A. No; a very moderate quantity of plain food is all that is necessary for the support of health and strength; all beyond this will injure both.

Q. 254. What are the effects produced by eating too much?

A. The powers of the stomach are impaired; the body becomes bloated, languid, and enfeebled, the spirits depressed, the mind inactive; finally the system becomes the prey of gout, dropsy, appoplexy, and other serious diseases, and the duration of life is considerably shortened.

Q. 255. What is necessary in order that wholesome food, eaten in moderation, may communicate a sufficient amount of nourishment to the system?

A. That the powers of the stomach be sufficient to its perfect digestion.

Q. 256. What are the causes by which the powers of the stomach are impaired?

A. By intemperance in eating, by drinking distilled spirits, or immoderate quantities of wine and malt liquors, by the neglect of exercise, by over fatigue, want of regular sleep, impure air, intense application of the mind, anxiety, &c.

Q. 257. How are the healthy powers of the stomach best preserved?

A. By drinking only water, by regular active exercise in the open air; by partaking of wholesome and simple food, plainly cooked; by eating only when an appetite is present, and leaving off the moment it is satisfied; by personal cleanliness; breathing a pure atmosphere, and by preserving the mind free from all corroding cares.

Q. 258. In what manner do the refinements of cookery, rich sauces and spices, become injurious?

A. They disorder the stomach, and impair the health of the system generally, by rendering the food too heating, and difficult of digestion, and by inducing us to partake of too much food, or to eat in the absence of the natural appetite.

Q. 259. Is it proper to partake of food while the body is laboring under the immediate effects of considerable fatigue?

A. Whatever food is then eaten should at least be very light, in a fluid form, and taken in moderation.

Q. 260. How many meals should an individual take in the course of the day?

A. On this subject, no rule can be laid down; the wants of the system as indicated by a healthy appetite should be the only guide as to the frequency and extent of our meals.

Q. 261. In a healthy condition of the system at what period will food be most generally required?

A. Soon after waking in the morning, and also towards the close of the day. Breakfast and an early supper would appear, therefore, to be the most indispensible meals to an individual in health and using daily active exercise.

Q. 262. Is it proper to allow the stomach to endure for any length of time the sensation of hunger?

A. No; by so doing, its powers are always impaired.

Q. 263. Who requires the most food, the individual who devotes the greater part of the day to bodily exercise, or he who spends it in sedentary employments within doors?

A. The first will require the most food; this is indicated by a greater appetite and stronger powers of digestion which they possess.

Q. 264. In which season of the year, summer or winter, is the greatest amount of food necessary?

A. In winter. In summer the food should consist principally of vegetables, of preparations of milk and the like.

Q. 265. Is it proper to drink a great quantity at meals?

A, No; drinking a large amount of fluid at meals, impedes the digestion of the food.

Q. 266. What constitutes an important article of food in all civilized countries?

A. Bread, which has been justly styled the staff of life.

Q. 267. What are the requisites to constitute bread a wholesome food?

A. That it be made of good flour, that the dough be rendered sufficiently light before it is put in the oven, and that it be well baked, and at least one day old before it is used.

Q. 268. Is not warm or perfectly fresh bread wholesome?

A. No; it is difficult of digestion, and always disorders the stomach.

Q. 269. Is bread that has been kept too long, or in a damp place, so as to become mouldy or musty, fit to be eaten?

A. No; such bread is liable to produce very serious disease.

Q. 270. Are hot cakes made of flour kneaded with butter or lard wholesome?

A. No;—when eaten they soon produce, particularly in weak stomachs, a sense of weight and uneasiness, and what is termed heart-burn.

Q. 271. Are potatoes a wholesome food?

A. When eaten with milk or with meat they are an excellent food, but they contain too little nourishment to permit the body to be supported on them alone.

Q. 272. Are very fat meats wholesome?

A. The fat of meat is always difficult of digestion, and when the individual is not very robust, it disorders the stomach to a very great extent.

Q. 273. Is tea a proper article of food?

A. Tea cannot be considered as an article of food, as it contains in itself not the least nourishment. When drank very strong or in large quantities, it undoubtedly injures the stomach and disorders the whole system.

Q. 274. Is coffee less injurious than tea?

A. From the moderate use of weak coffee, with plenty of sugar and milk, little or no injury can result; when very strong, however, or immoderately indulged in, it is predjudicial to health.

Q. 275. What effect has chocolate on health?

A. When perfectly pure, boiled in fresh milk, and drank in moderation, it is both wholesome and nourishing. The impure chocolate generally made use of, is, however, pernicious to health.

Q. 276. Is ripe fruit a wholesome article of food?

A. It is when eaten in moderation and with proper precautions.

Q. 277. What are those precautions?

A. Not to indulge in it when the stomach is loaded with other food; always to pare it or memove the external skin; and of plumbs, cherries and the like, always to reject the stones.

Q. 278. Why is it necessary to remove the skin and reject the stones of fruit?

A. Because the first being tough and indigestible, is liable to remain in the stomach for a long time, and to produce uneasiness and pain, and the latter, when swallowed, have been frequently the cause of most serious disease of the bowels, or even death.

Q. 279. Is unripe or decayed fruit unwholesome?

A. They are so in the highest degree.

Q. 280. Is fruit cooked with sugar injurious to the stomach?

A. Not when it is eaten in moderation, and the stomach is not laboring under disease.

Q. 281. Is milk a wholesome article of food?

A. Milk and all its simple preparations are among the most wholesome articles of food.

Q. 282. When we feel no appetite for food, cannot we excite one by taking bitters or cordials?

A. An artificial appetite may in this manner be excited, but the food that is then eaten, will not be perfectly digested, nor yield sufficient nourishment to the system, while the stomach is invariably injured.

Q. 283. When an individual has been so unwise as to overload the stomach with food what ought to be done?

A. He should abstain entirely for a short time from every kind of solid food, making use of a small quantity of toast water, thin gruel, or weak broth.

Q. 284. Are pie crust and the different kinds of pastry wholesome articles of food?

A. To weak stomachs and those of children they are in the highest degree injurious; to abstain entirely from their use is the wisest rule.

---

### MEANS OF SECURING THE SYMMETRY AND BEAUTY OF THE BODY.

Q. 428. Upon what is the physical beauty of man intimately dependant?

A. Upon the health, the full development, and the perfect conformation of his body.

Q. 429. In what manner may these be secured?

A. By the means which have been already pointed out; especially by temperance, and the free and regular exercise of the body in a pure air, commenced with in early life, and persevered in daily.

Q. 430. What are the means best calculated to secure that freshness and brilliancy of the complexion, regularity of features, and vivid expression of the eyes, so essential to the beauty of the face?

A. A free and pure air; regular exercise; washing daily with water, and frequent bathing; a light, easy dress, adapted to the temperature of the season; pure water for drink, and simple wholesome food to nourish the body.

Q. 431. By what means are the full growth and symmetrical development of the body promoted?

A. By avoiding sloth and inactivity, and too much confinement within doors, or in a sedentary posture: by rejecting all such articles of dress as bind, or press upon any part of the body: by avoiding gluttony, and every species of intemperance: and in our exercises, to shun all such as call into action only a part of the body, and leave the residue in a state of rest.

Q. 432. What is particularly demanded during youth, in order to secure the full and perfect development of the body?

A. That a considerable portion of each day be spent in childish sports and gymnastic exercises.

Q. 433. What position of the body during childhood and early life is best adapted to promote its growth and beauty?

A. The erect posture, whether in standing or walking; keeping the head and breast elevated, and resting the weight of the body equally upon both legs. On all occasions, therefore, that will admit of it, the upright posture is the one which should be insisted upon, in order to prevent permanent deformity.

Q. 434. What positions of the body are especially liable to occasion permanent deformity?

A. All such positions in which the body is bent to one side, or forwards, when continued for any length of time or frequently repeated; leaning both arms or the breast upon a table, when occupied in reading or writing; an habitual negligence in the carriage of the body, either when walking, standing, or sitting; hanging down the head, or bending the neck forwards, especially when speaking, or attentively listening; looking askance at objects, &c.

Q. 435. What important cautions with respect to the bed occupied by a young person are necessary in order to prevent deformity of the body?

A. To prevent the head and shoulders from being too much elevated by a large pillar or bolster, and to have the bed of sufficient firmness to prevent the hips from sinking into it.

Q. 436. What evil results when these circumstances are not attended to?

A. The back is very apt to become permanently twisted; one of the shoulders more elevated than the other, and the neck inclined ungracefully to one side.

Q. 437. Ought young people, when two sleep together, to be allowed always to occupy the same side of the bed?

A. No: they should change sides frequently; otherwise, particularly in a feather bed, they are liable to become crooked.

Q. 438. Is the same injury liable to result from sitting always at the same side of the table, or window, while at work?

A. It is. Young people, when the position in which they sit, is not freqnently changed, acquire a practice of bending habitually to one side, from which a permanent twist of the body in that direction is to be feared.

Q. 439. Has the foolish habit which many young persons indulge in, of contorting their features, or of shrugging their shoulders and performing similar gesticulations, any permanent influence upon their physical beauty?

A. All these practices, which are in the highest degree ungraceful, when frequently indulged in, are very apt to become habitual—that is, to be repeated involuntarily: when this is the case, the manner in which they injure the beauty of the individual will be readily understood.

Q. 440. May not these practices be acquired involuntarily?

A. They may: thus when a lively and active child is kept for many hours in a room, without being allowed to vary his posture, or when he is constrained to apply himself too long at one time to his books, he becomes restless, and is very liable to acquire involuntary twitchings of the face and limbs.

Q. 441. Is the habit of squinting ever acquired by improper practices?

A. It is often acquired by proper care not being taken to accustom infants and young children in viewing objects to exercise both the eyes equally, or by allowing them habitually to view objects placed too much on one side, and not in the direct line of vision.

Q. 442. May not an improper form of the cap worn by infants, cause them to squint?

A. It is very probable that when the cap worn in infancy projects forwards beyond the head on each side, like the blinds of a coach horse, so as to make it easier for the child as he lays in his cradle to view the objects on either side of him with the eye most distant from them, that squinting will be occasioned. It is at least important that the caps here described be carefully avoided.

---

### OF THE PASSIONS.

Q. 236. Has the proper government of the passions any connection with the preservation of health?

A. Yes, a very important one: unless the passions of the mind be kept in subjection, health invariably suffers.

Q. 237. What disposition of the mind is most conducive to health and longevity?

A. A calm, contented and cheerful disposition.

Q. 238. In what manner does the inordinate indulgence of the passions act prejudicially upon health?

A. It impairs indigestion; suspends the proper functions of the skin; injures the nervous system; disorders the circulation, either by inordinately exciting, or by depressing the action of the heart and blood vessels, and renders the body more susceptible to all the causes of disease.

Q. 239. What are the passions an indulgence in which is most prejudicial to health?

A. Anger, hatred, revenge, jealousy, fear, grief, and despair.

Q. 240. What persons are most subject to inordinate fits of anger?

A. They who are intemperate in eating and drinking; particularly such as indulge in the use of ardent spirits.

Q. 241. Has the indulgence of violent anger been known to produce immediate death?

A. It has in many instances. History furnishes us with numerous striking examples.

Q. 242. In whom is the indulgence of anger liable to be attended with the most fatal effects?

A. In those of full habits, with short, thick necks; in those labouring under disease of the heart or lungs, or who are subject to a spitting of blood, or to convulsions.

Q. 243. Has violent paroxysms of anger indulged in by nurses, any effect upon the infant to which they give suck?

A. Such a change is frequently produced in the milk by this cause, as to induce convulsions and even death in the infant, when applied to the breast during, or immediately after a violent fit of anger in the nurse.

Q. 244. Is the sudden destruction of life ever caused by excessive joy?

A. It is, though not so frequently as from violent bursts of anger.

Q 245. Has excessive fear ever been known to destroy life?

A. It has frequently caused instantaneous death; when this is not the case, it is apt to produce insanity, convulsions, or a state of fatuity.

Q. 246. What effect has extreme fear upon the hair?

A. It has been known to render it perfectly white, instantly, or in a very few hours.

Q. 247. Is not the same effect most generally produced by grief and anxiety?

A. It is, but not commonly in so short a period as by fear.

Q. 248. Does any particular mode of life increase the susceptibility of the mind to fear?

A. Yes—the susceptibility to fear is most usually increased by a luxurious and indolent mode of life; neglect of bodily exercise; or in fact, by what ever reduces the energies of the system.

Q. 249. Are there no instances, however, of persons in perfect health, and endowed with great bodily strength, being peculiarly subject to excessive fear?

A. Yes: ignorance, superstition and guilt will cause the mind to be disturbed by extreme terror from the most trifling causes, when neither the strength nor health of the body is impaired.

Q. 250. In sickly seasons, or during the prevalence of an epidemic, are persons who give way readily to fear, particularly liable to be attacked by disease?

A. Yes, even under a less degree of exposure than individuals of an opposite character. the same, however, is true of all who labor under the effects of any of the depressing passions.

Q. 251. Is the occurrence of any circumstance calculated to give the mind a sudden shock attended with danger?

A. On delicate and very sensible habits, it produces the same bad effects as excessive fear.

Q. 252. What effect has a paroxysm of any of the passions upon the appetite?

A. It will not only immediately destroy the appetite, but it will suspend also the digestive powers of the stomach, so that food taken under such circumstances will either remain unchanged or run into fermentation.

Q. 253. What influence has the inordinate indulgence of the passions upon the natural disposition to sleep?

A. It very generally suspends it. Persons laboring under the influence as well of the exciting as of the depressing passions, have been known to experience an inability to sleep for many nights.

Q. 254. Does the inordinate indulgence of the passions produce any effect upon the nutrition of the system?

A. It almost always diminishes it: hence the peevish, those laboring under sorrow, anxiety, disappointment, or whose minds are the seat of jealousy, hatred, or remorse, very generally become emaciated.

Q. 255. Is joy, when moderately indulged in, prejudicial to health?

A. On the contrary, it increases digestion, promotes the circulation of the blood, facilitates the healthy action of the skin, and acts as a beneficial stimulous upon the brain and whole nervous system.

Q. 256. Has the indulgence of hope any beneficial effect?

A. The anticipation of future good, or of some favorable change, when moderately indulged in, produces the same beneficial effects as moderate joy: but irrational, ungrounded, or misplaced hopes, on the other hand, are productive of injury, which is increased by the severe disappointment and chagrin to which their ultimate failure sooner or later gives rise.

Q. 257. Have not the hopes of a happy immortality the power of increasing our health and longevity?

A. There can be little doubt that the state of mind caused by the possession of such hopes acts beneficially upon the health, and may even prolong our days.

Q. 258. Is the studious cultivation of a kind and virtuous disposition influencial in promoting health?

A. It is in a very high degree. The fact cannot be too strongly impressed upon the mind of youth, that between vice and disease there exists an intimate connexion.

Q. 259. Is the indulgence of ambition injurious to the system?

A. Ambition, governed by discretion and directed to commendable objects, has a beneficial influence upon the system; but when inordinate, or tending to improper or unworthy ends, its effects are in the highest degree prejudicial.

Q. 260. When individuals are attacked with disease, in whom is the danger always the greatest?

A. In those whose minds are in a constant state of perturbation from the indulgence of passion, or are depressed by fear, grief, anxiety, remorse, or disappointment.

Q. 261. Are not such individuals, likewise, those most subject to diseases of the mind, and to the loss of reason?

A. It is unquestionably the case that in such nearly all the diseases of the mind, including mania, are of more frequent occurrence than in those whose passions are less active, or kept more perfectly under the control of reason.

Q. 262. In what manner does intense grief prove prejudicial to health?

A. By depressing the action of the brain and nerves, destroying the appetite, impairing digestion, and rendering defective the nutrition of the body.

Q. 263. While the state of the mind has so powerful an influence over the health of the system, is the mind in any degree affected by the state of the body?

A. The influence is reciprocal: a slight disturbance of the stomach from too much or indigestible food is sufficient often to render the temper irritable and peevish, or discontented with every thing to which its attention is directed.

---

### CONCLUSION.

Q. 264. From the preceding view of the causes by which the health and vigor of the system are promoted or impaired, what general conclusion is to be drawn?

A. That they who owe their birth and education to healthy, well informed, and industrious parents; they who from their earliest infancy have constantly breathed a pure, fresh, and dry air, aud have been allowed the free and natural motion of their limbs in daily exercise; they whose persons and apparel are always preserved strictly clean; who in regard to their meals observe moderation, order and simplicity, and drink nothing but pure water; they whose habitations are orderly, clean, dry, and well ventilated; they who have been accustomed from their youth to order, assiduity and industry; whose reason and virtue have been fortified and improved by early instruction and example; and who have been taught to fear God, love mankind, and do justice to all; they and they alone can enjoy continued health and happiness, and have a well grounded hope of prolonging their mental and physical powers to the latest period. D.

